# Correlates of body image in individuals with endometriosis: The role of body compassion and endometriosis-related symptoms

**DOI:** 10.1177/13591053251401720

**Published:** 2026-01-15

**Authors:** Leesa Van Niekerk, Cecilia Hoi Man Ng, Louise Gibson, Rebecca O’Hara, Antonina Mikocka-Walus, Kimberley Norris, Mathew Leonardi, Mike Armour, Subhadra Evans

**Affiliations:** 1 University of Tasmania, Hobart, Australia; 2 University of New South Wales, Sydney, NSW, Australia; 3 Flinders University, Adelaide, SA, Australia; 4 Deakin University, Geelong, VIC, Australia; 5 McMaster University, Hamilton, ON, Canada; 6 Western Sydney University, Penrith, NSW, Australia

**Keywords:** body image, body compassion, defusion, psychological wellbeing, endometriosis

## Abstract

Knowledge pertaining to the interplay between body image, body compassion, and endometriosis-related symptoms is limited. The current study aimed to elucidate the relationships between body compassion, body image, and endometriosis-related symptoms, and explore whether endometriosis-related factors or body compassion are significant correlates of body image. Individuals with self-reported symptomatic endometriosis (*n* = 261), aged 18 years and over, provided endometriosis-related information and completed the Body Attitude Test and Body Compassion Scale. Regression analyses determined that the presence nonmenstrual abdominal pain, lower ability to defuse from negative body-related thoughts, lower body-related acceptance, and higher levels of common humanity were significant correlates of body image, as measured by the Body Attitude Test, in the current endometriosis sample. Preliminary support is noted for the potential inclusion of compassion-focused interventions for addressing body image concerns in endometriosis, with nonmenstrual abdominal pain, dyspareunia, nausea, and bloating viewed as important symptoms for consideration.

## Introduction

Endometriosis is a chronic fibrotic inflammatory disease where endometrium-like glands and/or stroma are identified outside of the uterine cavity (Tomassetti et al., 2021). In line with the recommendation to understand the pervasive impacts of endometriosis, outside of pain and fertility management alone, research has increasingly focused on determining how endometriosis may influence body-related factors such as body image and body awareness (Facchin et al., 2018; Gonçalves et al., 2016). An increased understanding of the relationships between endometriosis and body image is important as findings indicate that a more positive body image is associated with greater HRQoL, psychosocial wellbeing, and pain coping (Calvi et al., 2024; Volker and Mills, 2022).

Body image incorporates cognitive, affective, and behavioral responses to the body and includes both bodily appearance and functionality (Pehlivan et al., 2022) and is particularly relevant for endometriosis where aspects of the disease and treatment are associated with appearance-related aspects (e.g. surgical scars, abdominal bloating) and functionality (e.g. fatigue, fertility concerns). Furthermore, as the onset of endometriosis-related symptoms typically occurs during adolescence and other periods of significant life transition (e.g. forming of friendships, commencing sexual relationships, entering the workforce; Missmer et al., 2021), alterations in body image are not unexpected.

Studies have highlighted differences in body image concerns between individuals with and without endometriosis, with those with the condition reporting greater weight dissatisfaction, appearance concerns, and lack of body familiarity (Melis et al., 2015; Van Niekerk et al., 2022). A lack of body familiarity, an aspect of body image, includes a sense of discomfort within one’s body, a sense of disconnection from the body, or a sense of threat and fear associated with bodily experiences (Probst et al., 1997). Volker and Mills (2022) explored the relationship between endometriosis-related pelvic pain and body image and found that individuals with endometriosis demonstrated significantly poorer body image than those without endometriosis, and that the level of pelvic pain was significantly negatively associated with body image in those with the disease.

Given the documented changes in body image associated with endometriosis, and the interplay between body image and psychological wellbeing in endometriosis (Geller et al., 2021; Pehlivan et al., 2022), compassion-focused therapies may be particularly relevant for inclusion in the treatment of endometriosis (Skinner and Kuijer, 2025). More specifically, compassion-focused interventions have been recommended to address body image concerns (e.g. low body familiarity) given the potential for positive body image to improve emotional and physical wellbeing in people living with endometriosis (Pehlivan et al., 2024; Van Niekerk et al., 2023).

Qualitative research has documented that participants report multiple impacts within the physical domain of self-identity, including a loss of trust in the body and a perception of the body being broken or failing (Mazalin et al., 2025; Mills et al., 2022). Altman et al. (2020) introduced the construct of body compassion to capture the multi-dimensional nature of the relationship to and experiences of the body. Body compassion incorporates Neff’s (2003) conceptualization of self-compassion with Cash and Fleming’s (2002) conceptualization of body image to directly measure compassion for the body, facilitating an understanding of how an individual relates to perceived physical limitations, inadequacies, or flaws (Altman et al., 2020). Body compassion provides a nuanced focus on the physical self and an individual’s capacity to engage compassionately with the body rather than being critical or fearful of endometriosis-related experiences (Altman et al., 2020; Mazalin et al., 2025). Body compassion research in populations where body-related concerns are evident has found that it may serve as a protective mechanism against body-related distress (Oliveira et al., 2018). Furthermore, assessing body compassion may facilitate a deeper understanding of the connection to the body as it navigates symptoms of endometriosis and endometriosis-related physical changes and how these experiences may influence body image.

A shift in focus from the general self to experiences grounded within the body is important for developing body-based endometriosis-related interventions aimed at enhancing compassion, given the physical embodiment of the disease (Altman et al., 2020). Theoretically, higher levels of body compassion would likely be associated with an increased ability to adjust and cope with endometriosis-related symptoms and a more positive body image. Research investigating body compassion and endometriosis is in its infancy, with body compassion found to be a stronger correlate of physical wellbeing, bodily pain, vitality, and social engagement than self-compassion alone (Van Niekerk et al., 2023). However, research examining the link between body compassion and body image in endometriosis is lacking. Therefore, the current study aimed to elucidate the relationships between body compassion, body image, and endometriosis-related factors in individuals with endometriosis. The study also aimed to explore whether individual endometriosis-related factors (e.g. disease duration, endometriosis-related pain) or body compassion are associated with variability in body image. These findings may assist in informing person-centered compassion-focused mind-body interventions and psychological treatment planning for endometriosis, with a focus on addressing alterations to physical self-concept associated with impaired body image.

## Method

### Participants

The sample consisted of 261 individuals, presumed female at birth, with self-reported symptomatic endometriosis. Participants were aged between 18 and 50 years of age (*M* = 31.28 years, SD = 7.83), with 252 participants identifying as women and 9 as nonbinary/gender queer/gender fluid. Inclusion criteria for participation were (1) a self-reported surgical or clinical diagnosis of endometriosis, (2) aged 18 years or older, and (3) have experienced a minimum of one endometriosis-related symptom in the 4 weeks prior to survey completion.

The participant sample was recruited via advertising at gynecology clinics, social media sites (EndoActive, EndoZone, Endometriosis Australia), and snowball sampling (April 2022 to December 2022). The online survey was presented on the Research Electronic Data Capture (REDCap) survey platform (Harris et al., 2009). Participants were provided with a detailed Participation Information Sheet outlining the purpose of the research, the voluntary nature of the research, and capacity to withdraw at any stage without explanation. Only fully completed and submitted surveys were included in data analysis. Ethical approval for the research was provided by the University of Tasmania Human Research Ethics Committee (H0026906). A subset of data examining the relationship between HRQoL, sexual functioning, and laparoscopic surgery has been previously published (Van Niekerk et al., 2024).

### Measures

#### Sociodemographic and endometriosis-related information

Sociodemographic and endometriosis-specific information was collected at the beginning of the online survey. Participants were asked to endorse (present/absent) specific endometriosis symptoms they had experienced in the 4-week period prior to survey completion. Participants also indicated the level of distress associated with the presence of each endorsed symptom (0 = No Distress to 3 = Extreme Distress; Van Niekerk et al., 2022; see Supplemental Table 1).

#### Body-related measures

The *Body Compassion Scale* (BCS; Altman et al., 2020) is a 23-item scale, anchored in mindfulness and acceptance, bridging self-compassion and body image, and provides a measure of compassion an individual holds toward their body. The scale consists of three factors: defusion, common humanity, and acceptance. Respondents rate their body-related beliefs and behaviors on a 5-point Likert-type scale (1 = Almost Never to 5 = Almost Always), with overall body compassion scores ranging from 26 to 113, with higher scores indicating greater body compassion. The BCS has sound convergent validity (Policardo et al., 2022), and excellent internal consistency (Van Niekerk et al., 2023a).

The *Body Attitude Test* (BAT; Probst et al., 1995) is a 20-item self-report questionnaire that assesses respondents’ body-based attitudes and beliefs. Respondents rate their level of agreement for each statement on a 6-point Likert-type scale (0 = Never, 5 = Always). The BAT has three factor scores of negative appreciation of body size, lack of familiarity with one’s own body, and general body dissatisfaction. Higher scores reflect a more negative attitude toward one’s body, with a clinical cut off score of 36 or greater indicating clinically impaired body image. The BAT has been found to be a valid and reliable measure of subjective body experiences and attitude toward the body in both clinical and nonclinical samples (Probst et al., 2009) and has been used to measure body image in gynecological disorders including endometriosis and polycystic ovary syndrome (Melis et al., 2015; Van Niekerk et al., 2022, 2022a) and where body image may be impacted by health diagnoses such as breast cancer (Şahin et al., 2022).

### Design and data analysis

A cross-sectional design was utilized to determine the presence, degree, and direction of associations between endometriosis-related variables, body compassion, and body image. A priori power analysis indicated a minimum of 208 responses were required for linear regression models to achieve a power of 0.95, *p* = 0.05, indicating that the final sample of 228 responses provides sufficient power. Spearman’s Rho bivariate correlations were completed to determine the associations between distress associated with the presence of endometriosis-related symptoms, body compassion, and body image. Hierarchical multiple regression analyses (MRA) were conducted to explore the unique contribution that individual endometriosis-related factors (block one), the presence/absence of endometriosis-related symptoms (block two), and body compassion (block three) have in influencing body image for individuals with endometriosis. Multicollinearity analyses indicated that both the Variance Inflation Factor (VIF) and Tolerance Scores (TS) were within acceptable ranges (VIF ⩽ 3 and TS ⩾ 1), except for endometriosis-related symptom burden (VIF ⩾ 5), so this variable was excluded from the regression analyses. Statistical analyses were conducted using SPSS 24. Statistical significance was set at *p* ⩽ 0.05.

## Results

Most of the participants resided in the Oceania region (88.5%), were in a committed relationship or married (73.6%), heterosexual (73.6%), and non-menopausal (79.3%), with 10.4% experiencing surgical or medical menopause. Menopausal status was not found to significantly influence the study outcomes (see Supplemental Results File 1). The mean BMI for the sample was 27.08 (*SD* = 6.15, range = 16.26–47.27). Further participant characteristics are outlined in Table 1.

**Table 1.** Participants sociodemographic characteristics.

Demographic characteristic	Frequency	Percent (%)
Country of residence		
Australia/Oceania (e.g., New Zealand, Papua New Guinea)	231	88.5
United Kingdom	10	3.8
North America	20	7.7
Highest level of academic attainment		
High school or below	42	16.1
Vocational certificate/qualification	55	21.1
Bachelor/Postgraduate degree	164	62.8
Employment status		
Student	51	19.5
Employed	166	63.6
Unable to work due to health	46	16.9
Relationship status		
Single	69	26.4
Committed	111	42.5
Married	81	31.1
Sexuality		
Gay, lesbian, or same gender attracted	22	9.2
Straight/heterosexual	192	73.6
Bisexual	34	13.0
Asexual	13	4.9
Reproductive history		
I have never tried to conceive	155	59.4
I have tried to conceive but have never been pregnant	23	8.8
I am currently trying to conceive	15	5.7
I am currently pregnant	2	0.8
I have been pregnant/no miscarriages	23	8.8
I have been pregnant/1or more births/1or more miscarriages	24	9.2
I have been pregnant/Nil live births	19	7.3

Approximately half of the sample (53.6%) self-reported a disease stage of moderate (stage III) to severe (stage IV). The sample reported a mean endometriosis-related symptom duration of 14.1 years (SD = 7.6) and experienced an average of 8.8 endometriosis-related symptoms (*SD* = 3.1, range = 1–16 symptoms) in the 4 weeks prior to completing the survey. On average, the endorsed endometriosis-related symptoms were rated as moderately distressing (*M* = 1.8, *SD* = 0.5). The sample reported a mean endometriosis-related pain level of 5.5 in the previous 7-day period (0 = No pain, 10 = Worst pain imaginable; *SD* = 2.5, range = 0–10) and most reported a minimum of one laparoscopic endometriosis-related surgery (87.4%; range = 0–9 surgeries). BMI was not significantly correlated with endometriosis-related symptom burden (*r* = 0.01, *p* = 0.848), symptom distress (*r* = −0.05, *p* = 0.422) or pain level (*r* = 0.11, *p* = 0.085).

### Body-related outcomes

#### Body Compassion Scale (n = 228)

On average, a moderate level of total body compassion was noted in the current sample (*M* = 58.6, SD = 14.2, range = 26–114). Moderate levels of defusion (*M* = 22.0, SD = 7.4, range = 9–45), common humanity (*M* = 23.4, SD = 7.5, range = 9–44), and acceptance (*M* = 13.1, SD = 4.5, range = 5–25) were also found. BMI was found to be statistically significantly negatively correlated with total body compassion, defusion, and acceptance, with small to medium effect size, indicating higher BMI is associated with lower levels of body compassion, lower ability to defuse from negative body-related experiences, and lower acceptance of the body (see Table 2).

**Table 2.** Associations between the distress associated with individual endometriosis-related symptoms, body compassion, and body image.

Variable	1.	2.	3.	4.	5.	6.	7.	8.	9.	10.	11.	12.	13.	14.	15.	16.	17.	18.	19.	20.	21.	22.	23
1. Dysmenorrhea	-	0.001	0.001	0.637	0.175	0.140	0.157	0.001	0.078	0.466	0.305	0.095	0.013	0.177	0.008	0.011	0.674	0.009	0.118	0.217	0.015	0.017	0.168
2. Back pain	**0.36**	-	0.001	0.002	0.032	0.001	0.165	0.001	0.102	0.001	0.001	0.005	0.001	0.036	0.286	0.003	0.058	0.341	0.006	0.001	0.055	0.001	0.103
3. Abdominal pain	**0.36**	**0.47**	-	0.011	0.009	0.001	0.003	0.182	0.835	0.001	0.001	0.001	0.007	0.483	0.004	0.001	0.459	0.009	0.003	0.001	0.012	0.001	0.175
4. Dyspareunia	0.06	**0.32**	**0.25**	-	0.001	0.008	0.046	0.110	0.857	0.364	0.806	0.005	0.002	0.003	0.931	0.014	0.002	0.230	0.040	0.037	0.015	0.004	0.252
5. Post coital	0.20	**0.25**	**0.29**	**0.60**	-	0.157	0.045	0.011	0.010	0.001	0.315	0.006	0.001	0.001	0.243	0.002	0.447	0.816	0.015	0.555	0.011	0.004	0.177
6. Bowel pain	0.15	**0.26**	**0.30**	**0.27**	0.16	-	0.001	0.004	0.084	0.003	0.102	0.001	0.057	0.424	0.589	0.004	0.065	0.969	0.058	0.002	0.097	0.006	0.033
7. Urination pain	0.16	0.16	**0.33**	**0.29**	**0.31**	**0.46**	-	0.001	0.488	0.216	0.100	0.006	0.014	0.549	0.837	0.963	0.190	0.313	0.845	0.174	0.192	0.487	0.306
8. HMB	**0.47**	**0.44**	0.16	0.26	**0.49**	**0.38**	**0.50**	-	0.766	0.008	0.077	0.118	0.296	0.082	0.949	0.837	0.033	0.144	0.480	0.676	0.117	0.353	0.833
9. Fertility	0.38	0.33	0.04	0.01	**−0.71**	−0.39	−0.25	−0.08	-	0.847	0.060	0.018	0.897	0.856	0.689	0.929	0.712	0.389	0.520	0.068	0.381	0.838	0.973
10. Fatigue	0.07	**0.33**	**0.29**	0.10	**0.43**	**0.24**	0.14	**0.32**	0.04	-	0.001	0.001	0.018	0.278	0.370	0.109	0.854	0.439	0.739	0.007	0.126	0.101	0.108
11. Bloating	0.10	**0.33**	**0.29**	0.03	0.11	0.13	0.18	0.21	0.38	**0.32**	-	0.006	0.001	0.637	0.152	0.128	0.276	0.020	0.014	0.017	0.036	0.001	0.325
12. Nausea	0.19	**0.24**	**0.33**	**0.31**	**0.33**	**0.29**	**0.33**	0.21	**−0.60**	**0.27**	**0.23**	-	0.010	0.433	0.063	0.017	0.435	0.002	0.048	0.001	0.001	0.001	0.615
13. Vulva pain	**0.42**	**0.40**	**0.35**	**0.47**	**0.52**	0.26	**0.45**	0.23	0.01	0.21	**0.48**	**0.35**	-	0.001	0.413	0.002	0.058	0.020	0.001	0.081	0.001	0.001	0.071
14. Clitoral pain	0.44	**0.46**	0.16	**0.76**	**0.93**	0.20	0.19	0.65	0.01	0.26	0.12	−0.19	**0.73**	-	0.040	0.096	0.481	0.143	0.051	0.098	0.014	0.005	0.009
15. Body Compassion Scale Total	**−0.25**	−0.08	**−0.21**	−0.09	−0.12	−0.04	0.02	0.08	−0.08	−0.07	−0.10	−0.15	−0.10	**−0.43**	-	0.001	0.001	0.001	0.001	0.001	0.001	0.001	0.003
16. BCS defusion	**−0.23**	**−0.22**	**−0.26**	**−0.23**	**−0.33**	**−0.22**	−0.01	−0.03	0.02	−0.12	−0.11	**−0.19**	**−0.37**	−0.36	**0.76**	-	0.037	0.001	0.001	0.001	0.001	0.001	0.050
17. BCS common humanity	−0.04	0.14	0.05	**0.29**	0.08	0.14	0.14	**0.25**	−0.07	0.01	0.08	0.06	0.24	−0.16	**0.65**	**0.14**	-	0.001	0.341	0.664	0.863	0.117	0.556
18. BCS acceptance	**−0.24**	−0.07	**−0.19**	−0.11	−0.03	0.01	−0.11	−0.17	−0.17	−0.06	**−0.17**	**−0.25**	**−0.29**	−0.32	**0.74**	**0.55**	**0.24**	-	0.001	0.001	0.001	0.001	0.001
19. BAT NABS	0.15	**0.21**	**0.21**	**0.19**	**0.26**	0.15	−0.02	0.08	−0.12	0.02	**0.18**	**0.16**	**0.41**	0.41	**−0.51**	**−0.58**	−0.03	**−0.66**	-	0.001	0.001	0.001	0.001
20. BAT LBF	0.12	**0.32**	**0.32**	**0.19**	0.06	**0.23**	−0.14	0.05	0.34	**0.19**	**0.17**	**0.27**	0.22	0.35	**−0.35**	**−0.51**	−0.01	**−0.28**	**0.41**	-	0.001	0.001	0.395
21. BAT Dissatisfaction	**0.22**	0.14	**0.18**	**0.22**	**0.27**	0.13	0.14	0.18	−0.17	0.11	**0.15**	**0.28**	**0.41**	**0.51**	**−0.65**	**−0.65**	−0.14	**−0.81**	**0.80**	**0.38**	-	0.001	0.001
22. Body Attitude Test Total	**0.22**	**0.25**	**0.27**	**0.26**	**0.30**	**0.21**	0.07	0.11	−0.04	0.12	**0.24**	**0.27**	**0.45**	**0.57**	**−0.61**	**−0.69**	−0.06	**−0.72**	**0.92**	**0.61**	**0.90**	-	0.001
23. Body Mass Index	0.12	0.12	−0.09	−0.10	−0.13	**−0.15**	−0.10	−0.02	0.01	−0.11	−0.07	−0.04	0.21	**0.50**	**−0.18**	**−0.12**	−0.04	**−0.30**	**0.46**	0.05	**0.39**	**0.37**	-

*Note*. Significance level (two-tailed) reported in upper half of matrix. Spearman Rho correlation presented in bottom half of matrix. Significant correlations in bold font (*p* < 0.05–*p* < 0.001).

Back pain: lower back pain when not menstruating; Abdominal pain: abdominal pain when not menstruating; Post coital: pain after sexual intercourse; HMB: heavy menstrual bleeding; Fertility: difficulty conceiving; BCS Defusion: body compassion defusion; BCS Common Humanity: body compassion common humanity; BCS Acceptance: body compassion acceptance; BAT NABS: body attitude test negative appreciation of body size; BAT LBF: body attitude test lack of body familiarity; BAT Dissatisfaction: Body attitude test body dissatisfaction;

#### Body attitude test (n = 228)

The total BAT score (*M* = 50.5, SD = 15.5, range = 14–89) was above the clinical cut off score of 36 for nonclinical populations and consistent with the mean score for Anorexia samples (*M* =50.4, *SD* = 16.5; Probst et al., 1995), indicating a level of body image impairment more consistent with a clinical than nonclinical sample. The sample reported a moderate level of negative appreciation of body size (*M* = 18.0, SD = 7.2, range = 2–35), lack of body familiarity (*M* = 14.6, SD = 4.7, range = 5–31), and general body dissatisfaction (*M* = 11.8, SD = 5.13, range = 0–20). BMI was statistically significantly correlated with total BAT score, negative appreciation of body size, and general body dissatisfaction, indicating that higher BMI is associated with a more negative attitude toward the body, a more negative view of body size, and greater general body dissatisfaction (see Table 2).

#### Body compassion, body image, and endometriosis-related symptoms

As can be seen in Table 2, the level of distress associated with the presence of certain endometriosis-related symptoms was found to be statistically significantly associated with body compassion and body image. Defusion was found to be negatively correlated with the level of distress associated with the presence of dysmenorrhea, lower back and abdominal pain (when not menstruating), dyspareunia, pain following sexual intercourse or associated with bowel movements, nausea, and vulvar pain, with small to medium effect sizes. This indicates that a lowered ability to defuse from negative body-related thoughts and emotions is linked to higher levels of distress associated with the presence of these symptoms. Acceptance was found to be negatively correlated with the level of distress associated with dysmenorrhea, abdominal pain (when not menstruating), bloating, nausea, and vulvar pain, with small effect sizes. This indicates that a lower level of body-related acceptance is linked to higher levels of distress associated with the presence of these symptoms. Common humanity was found to be negatively correlated with the level of distress associated with dyspareunia and heavy menstrual bleeding, with small effect sizes. This indicates that a greater sense of common humanity is linked to lower levels of distress associated with the presence of these symptoms.

Negative appreciation of body size was found to be statistically significantly positively correlated with the level of distress associated with the presence of lower back and abdominal pain (when not menstruating), dyspareunia, pain following sexual intercourse, bloating, nausea, and vulva pain, with small to medium effect sizes. This indicates that a greater negative appreciation of body size is linked to higher levels of distress associated with the presence of these symptoms. Lack of body familiarity was found to be positively correlated with the level of distress associated with the presence of lower back and abdominal pain (when not menstruating), dyspareunia, bowel pain, fatigue, bloating, and nausea, with small effect sizes. This indicates that a greater lack of body familiarity is linked to higher levels of distress associated with the presence of these symptoms. General body dissatisfaction was found to be positively correlated with the level of distress associated with the presence of dysmenorrhea, abdominal pain (when not menstruating), dyspareunia, pain following sexual intercourse, bloating, nausea, vulvar pain, and clitoral pain, with small to medium effect sizes. This indicates that greater general body dissatisfaction is linked to higher levels of distress associated with the presence of these symptoms.

### Contribution of endometriosis-specific factors, endometriosis-related symptoms, and body compassion to body image

Hierarchical MRA was used to determine the unique contribution of the presence of endometriosis-related factors, the presence of endometriosis-related symptoms, and body compassion on body image outcomes. Separate models were analyzed for negative appreciation of body size, lack of body familiarity, and general body dissatisfaction to determine whether differences exist in different body image domains.

#### Negative appreciation of body size

On Step 1 of the hierarchical MRA, endometriosis-related factors accounted for a significant 19.7% of the variance in negative appreciation of body size, *F*(5, 222) = 10.9, *p* = 0.001. On Step 2, endometriosis-related symptoms accounted for a significant additional 7.7% of the variance in negative appreciation of body size, *R*^2^ = 0.274, *F*(9, 213) = 2.5, *p* = 0.009. On Step 3 of the model, body compassion factors accounted for a significant 36.3% additional variance in negative appreciation of body size, *R*^2^ = 0.637, *F*(3, 210) = 69.9, *p* = 0.001. In combination, the correlates accounted for 63.7% of the variance, *R*^2^ = 0.637, adjusted *R*^2^ = 0.608, *F*(17, 210) = 21.7, *p* = 0.001.

As can be seen in Table 3, the significant correlates of negative appreciation of body size in the final model were BMI, symptom duration, the presence of abdominal pain, defusion, common humanity, and acceptance, with higher BMI, shorter disease duration, the presence of nonmenstrual abdominal pain, lower ability to defuse from negative body-related thoughts and emotions, lower body-related acceptance and higher levels of common humanity associated with greater negative appreciation of body size in the current sample.

**Table 3.** Correlates of body image domains in individuals with endometriosis.

Correlate	Negative appreciation				Lack of familiarity				General dissatisfaction			
	*b*	*β*	*t*	*p*	*b*	*β*	*t*	*p*	*b*	*β*	*t*	*p*
Step 1												
Age	0.03	0.03	0.45	0.653	−0.03	−0.05	−0.69	0.488	−0.01	−0.01	−0.08	0.937
Body Mass Index	0.37	0.35	5.64	**0.001**	−0.02	−0.03	−0.48	0.629	0.21	0.28	4.46	**0.001**
Symptom duration	−0.09	−0.10	−1.16	0.247	0.02	0.03	0.03	0.973	−0.03	−0.05	−0.63	0.526
Symptom distress	2.19	0.16	2.30	**0.022**	2.34	0.26	3.77	**0.001**	2.33	0.23	3.37	**0.001**
Pain VAS	0.03	0.12	1.79	0.073	0.05	0.26	3.83	**0.001**	0.01	0.05	0.75	0.449
Step 2												
Age	0.08	0.09	1.04	0.297	0.01	0.001	0.013	0.989	0.01	0.02	0.27	0.785
Body Mass Index	0.38	0.36	5.87	**0.001**	−0.03	−0.04	−0.74	0.458	0.20	0.27	4.35	**0.001**
Symptom duration	−0.17	−0.18	−2.25	**0.025**	−0.02	−0.03	−0.40	0.686	−0.07	−0.11	−1.34	0.179
Symptom distress	2.80	0.20	2.87	**0.004**	1.88	0.20	2.88	**0.004**	2.70	0.27	3.77	**0.001**
Pain VAS	0.01	0.05	0.77	0.438	0.04	0.21	2.77	**0.006**	0.01	0.02	0.26	0.791
Dysmenorrhea	−1.99	−0.14	−1.71	0.088	−0.46	−0.04	−0.59	0.555	−1.48	−0.14	−1.73	0.085
Abdominal	2.87	0.15	2.27	**0.024**	−0.15	−0.01	−0.18	0.852	1.07	0.07	1.15	0.250
Bowel pain	1.77	0.11	1.47	0.141	−0.02	−0.02	−0.03	0.973	2.05	0.17	2.33	**0.020**
Urination pain	−1.67	−0.11	−1.78	0.077	0.27	0.02	0.44	0.659	−2.34	−0.22	−3.39	**0.001**
HMB	−0.24	−0.02	−0.19	0.847	1.45	0.14	1.67	0.095	0.468	0.04	0.49	0.622
Constipation	0.82	0.05	0.77	0.439	−0.04	−0.04	−0.05	0.953	−0.04	−0.01	−0.05	0.954
Bloating	0.96	0.05	0.74	0.461	1.33	0.10	1.54	0.125	0.64	0.04	0.67	0.502
Vulva	−0.99	−0.06	−0.89	0.374	1.25	0.12	1.68	0.094	−0.69	−0.06	−0.85	0.394
Clitoral	3.89	0.16	2.36	**0.019**	0.39	0.02	0.36	0.719	1.46	0.08	1.21	0.227
Step 3												
Age	0.11	0.12	1.96	0.051	0.03	0.05	0.62	0.534	0.04	0.06	1.20	0.233
Body Mass Index	0.27	0.25	5.57	**0.001**	−0.06	−0.09	−1.50	0.135	0.10	0.13	3.60	**0.001**
Symptom duration	−0.13	−0.13	−2.22	**0.028**	−0.01	−0.01	−0.13	0.894	−0.04	−0.05	−1.09	0.278
Symptom distress	0.59	0.04	0.83	0.409	1.22	0.14	2.02	**0.045**	0.74	0.08	1.83	0.068
Pain VAS	−0.01	−0.04	−0.64	0.523	0.02	0.13	1.77	0.075	−0.01	−0.07	−1.60	0.110
Dysmenorrhea	−0.84	−0.06	-1.00	0.319	0.07	0.01	0.10	0.921	−0.58	−0.06	−1.23	0.220
Abdominal	3.48	0.18	3.84	**0.001**	0.29	0.02	0.38	0.702	1.51	0.11	2.95	**0.004**
Bowel pain	−0.37	−0.02	−0.43	0.672	−0.74	−0.07	−1.01	0.316	0.12	0.01	0.24	0.812
Urination pain	0.46	0.03	0.65	0.517	0.75	0.08	1.30	0.210	−0.45	−0.04	−1.13	0.262
HMB	−0.73	−0.05	−0.80	0.427	1.02	0.10	1.31	0.193	0.15	0.01	0.29	0.775
Constipation	0.20	0.01	0.27	0.791	−0.19	−0.02	−0.29	0.772	−0.68	−0.06	−1.59	0.114
Bloating	−0.22	−0.01	−0.24	0.813	0.70	0.05	0.88	0.380	−0.23	−0.02	−0.45	0.656
Vulva	−0.36	−0.02	−0.45	0.655	1.01	0.10	1.47	0.142	0.08	0.01	0.18	0.859
Clitoral	2.30	0.10	1.91	0.058	−0.07	−0.01	−0.06	0.949	0.19	0.01	0.27	0.785
Defusion	−0.32	−0.32	−5.97	**0.001**	−0.25	−0.40	−5.68	**0.001**	−0.20	−0.29	−6.80	**0.001**
Humanity	0.11	0.11	2.32	**0.021**	0.02	0.04	0.63	0.528	0.06	0.09	2.38	**0.018**
Acceptance	−0.75	−0.47	−8.59	**0.001**	−0.08	−0.07	−1.05	0.296	−0.74	−0.66	−15.11	**0.001**

*Note*. Significant correlates noted in Bold font.

Symptom Duration: number of years experiencing endometriosis-related symptoms; Symptom Distress: average level of distress associated with the experience of endometriosis-related symptoms; Pain VAS: average level of endometriosis-specific pain experienced in the last 7-day period; HMH: heavy menstrual bleeding; Vulva: vulva pain; Clitoral: clitoral pain; Defusion: body compassion factor score (BCS); Humanity: common humanity Body compassion factor score (BCS); Acceptance: Body compassion factor score (BCS).

#### Lack of body familiarity

On Step 1 of the hierarchical MRA, endometriosis-related factors accounted for a significant 20.4% of the variance in lack of body familiarity, *F*(5, 222) = 11.4, *p* = 0.001. On Step 2, the presence of endometriosis-related symptoms accounted for a nonsignificant additional 3.6% of the variance in lack of body familiarity, *R*^2^ = 0.240, *F*(9, 213) = 1.1, *p* = 0.355. On Step 3 of the model, body compassion factors accounted for a significant 15% additional variance in lack of body familiarity, *R*^2^ = 0.390, *F*(3, 210) = 17.25, *p* = 0.001. In combination, the correlates accounted for 39% of the variance, *R*^2^ = 0.390, adjusted *R*^2^ = 0.341, *F*(17, 210) = 7.9, *p* = 0.001.

As can be seen in Table 3, the significant correlates of lack of body familiarity in the final model were endometriosis-related symptom distress and defusion, with higher levels of endometriosis-related symptom distress and lower ability to defuse from negative body-related thoughts and emotions associated with greater lack of body familiarity in the current sample.

#### General body dissatisfaction

On Step 1 of the hierarchical MRA, endometriosis-related factors accounted for a significant 15.3% of the variance in general body dissatisfaction, *F*(5, 222) = 8.2, *p* = 0.001. On Step 2, the presence of endometriosis-related symptoms accounted for a significant additional 6.9% of the variance in general body dissatisfaction, *R*^2^ = 0.225, *F*(9, 213) = 2.1, *p* = 0.030. On Step 3 of the model, body compassion factors accounted for a significant 54.7% additional variance in general body dissatisfaction, *R*^2^ = 0.772, *F*(3, 210) = 167.68, *p* = 0.001. In combination, the correlates accounted for 77.2% of the variance, *R*^2^ = 0.772, adjusted *R*^2^ = 0.753, *F*(17, 210) = 41.77, *p* = 0.001.

As can be seen in Table 3, the significant correlates of general body dissatisfaction in the final model were participant BMI, abdominal pain, defusion, common humanity, and acceptance, with higher BMI, the presence of nonmenstrual abdominal pain, lower ability to defuse from negative body-related thoughts and emotions, lower body-related acceptance and higher levels of common humanity associated with greater general body dissatisfaction in the current sample.

## Discussion

The current study aimed to elucidate the relationships between body compassion, body image, and endometriosis-related symptoms, and to explore whether endometriosis-related factors (e.g. disease duration, endometriosis-related pain) or body compassion are associated with variability in body image in individuals with endometriosis. Expanding past previous findings regarding endometriosis and body image (Sullivan-Myers et al., 2023), the current study found that body compassion and certain endometriosis-related symptoms are important considerations in understanding impaired body image in individuals living with the condition.

Understanding and addressing potential changes in body image for individuals with endometriosis is important given that Sullivan-Myers et al. (2023) found in their longitudinal study (*n* = 584) that a negative perception of one’s own body was a significant correlate of psychological distress in individuals with endometriosis. Furthermore, recent evidence suggests that impaired body image not only contributes to overall psychological distress but can also contribute to social loneliness among individuals with endometriosis (Calvi et al., 2024), and difficulty adjusting to the physical limitations imposed by the condition (Mills et al., 2023). These findings suggest that treatment focus is not limited to symptom reduction alone but also addresses the individual’s perceived level of distress associated with their endometriosis-related symptom profile.

In the current study, greater endometriosis-related pain was correlated with greater negative appreciation of body size, lack of body familiarity, and general body dissatisfaction. The finding that endometriosis-related pain may influence body image adds to previous research by Volker and Mills (2022), who also found a significant positive link between pain level and body image impairment, and Melis et al. (2015) who found that endometriosis-related pain was associated with a lack of body familiarity and general body dissatisfaction. The consistent finding of lowered levels of body familiarity in individuals with endometriosis is noteworthy given that a lack of body familiarity may significantly influence physical and emotional wellbeing (Van Niekerk et al., 2022). Furthermore, the current finding suggests that the level of endometriosis-related pain may be an important area of clinical focus in understanding and addressing body image (Melis et al., 2015). However, the current findings also indicate that a sole focus on pain management alone may not adequately address the key symptoms that influence body image, with body compassion highlighted as a potential area for clinical consideration. Furthermore, although endometriosis-related pain was found to be associated with body image concerns, it was not found to be a significant correlate in any of the final models of body image in the current endometriosis sample, with the body compassion factors of defusion, common humanity, and acceptance being found to be stronger correlates of body image in the current sample.

The distress associated with several individual endometriosis-related symptoms was found to be significantly associated with body image in the current sample. Particularly influential symptoms included the presence of nonmenstrual abdominal pain, nausea, dyspareunia, and bloating. Higher levels of distress associated with the presence of nonmenstrual abdominal pain, dyspareunia, nausea and bloating were associated with greater negative appreciation of body size, lack of body familiarity, and general body dissatisfaction. Furthermore, the presence of nonmenstrual abdominal pain was found to be a significant correlate of greater negative appreciation of body size and general dissatisfaction in the current sample. Abdominal pain has been found to influence pain-related disability in endometriosis (Evans et al., 2021) and nonmenstrual cramping has been found to be significantly associated with lowered HRQoL (Soliman et al., 2020). As 85% of the sample reported experiencing nonmenstrual abdominal pain and 66% reported experiencing nausea and their presence and associated level of distress were associated with impairments in body image, clinical assessment and management of abdominal pain and nausea, either as a primary symptom of endometriosis or a consequence of medication management, is important. Similarly, the significant finding relating to dyspareunia and body image emphasizes the importance of practitioners extending their focus beyond the link between dyspareunia and sexual function to understanding its influence more broadly (Grano et al., 2025).

Building on previous findings (Van Niekerk et al., 2023), the body compassion domains of defusion and acceptance were found to be significantly associated with body image, with a greater ability to defuse from negative body-related thoughts and emotions associated with more positive body image. Of note, the body compassion factor of defusion was found to be a significant negative correlate across all body image domains in individuals with endometriosis. As previous research has found that individuals with endometriosis may attempt to cope with symptoms such as pain by distancing themselves from their body (Melis et al., 2015), and that individuals with endometriosis experience lower levels of body familiarity (Van Niekerk et al., 2022), increasing engagement in defusion over distraction or dissociation may lead to positive improvements in psychological wellbeing and body image outcomes.

In contrast to distraction and dissociation, which result in a disconnection of the present moment and the body, defusion involves the ability to notice one’s thoughts (e.g. negative body and/or endometriosis-related thoughts) and see them as words, labels, or images rather than facts of statements of truth, allowing the person to stay connected to the present moment and their body (Yadavaia and Hayes, 2012), potentially increasing the person’s sense of body familiarity and reducing fear of endometriosis-related bodily experiences. Preliminary support for this hypothesis is centered on the current finding that a greater ability to engage in defusion was linked to greater body familiarity in the current sample. Further support can be seen in the emerging research investigating fear of disease progression and imagery in endometriosis. Todd et al. (2023) found preliminary evidence indicating a relationship between intrusive endometriosis-related images, fear of disease progression and pain interference, with individuals who are more distressed by their imagined endometriosis-related images having greater fear of disease progression and pain interference. Learning to defuse from negative endometriosis-related thoughts, emotions, and images to improve body awareness and familiarity may not only buffer against poor body image but also provide an avenue for managing fear of disease progression, pain-related interference, and anxiety associated with pelvic-based examinations or treatments (Arena et al., 2021).

The component of body-related acceptance may also have particular relevance for individuals with endometriosis in addressing body image concerns, with greater levels of body-related acceptance found to be a significant correlate of negative appreciation of body size and general body dissatisfaction in the current sample and linked to significantly lower levels of distress associated with nonmenstrual abdominal pain, nausea, vulva pain, and bloating. Previous research has found that greater psychological acceptance was a significant correlate of psychological wellbeing in individuals with endometriosis (*n* = 169), with researchers concluding that the capacity to experience pain, difficult thoughts, and emotions is more important than specific coping styles (Bernini et al., 2022). The current findings relating to body-related acceptance also highlight the possible benefit of acceptance-related psychological interventions for endometriosis, particularly for symptoms such as bloating that has been associated with body checking and hypervigilance to changes in bodily appearance (Mills et al., 2023). Interventions that incorporate acceptance practices focused on endometriosis-related pain and other aspects of living with endometriosis (i.e. fertility concerns, altered self-concept) may assist in improving psychological wellbeing and body image and the distress associated with symptoms such as bloating (Bernini et al., 2022; Kalfas et al., 2022).

The finding that a greater sense of common humanity is a significant correlate of greater negative appreciation of body size and general body dissatisfaction is unexpected given that the other body compassion factors (i.e. defusion, acceptance) appear to provide a buffer against body image concerns. This may reflect a similar finding regarding the role of common humanity in addressing body image concerns in non-endometriosis samples where common humanity was viewed as important by participants but not seen as a mechanism to cope with body image concerns (Mills et al., 2022; Seekis et al., 2022). Seekis et al. (2022) suggest that, while there may be some shared understanding that others experience body-related concerns, there may be a difference in acknowledging shared experiences compared to using this knowledge in a way that is self-protective. This finding may also be related to the outcome measures employed in the current study, with the Body Compassion Scale reflecting the respondent’s perception of shared experiences while the Body Attitude Test focuses on the individual experience of body-related concerns (Van Niekerk et al., 2023). While individuals diagnosed with endometriosis may acknowledge that there are shared body-related thoughts and emotions associated with the condition, this may increase body-related comparisons and a more negative perception of one’s own body.

The current findings indicate the importance of understanding endometriosis-related body-related concerns beyond body size (BMI) alone. While BMI was found to be a significant correlate of negative appreciation of body size and general body dissatisfaction in the current sample, it was not the strongest correlate in the final models. The presence of abdominal pain when not menstruating, the ability to defuse from negative body-related experiences, and acceptance were noted to be stronger correlates of the body image domains. This supports previous qualitative research reporting body-related distress associated with the presence of surgical scares and abdominal bloating rather than on body size alone (Sayer-Jones and Sherman, 2023). The importance of understanding body image beyond body size is further supported by Pellizzer et al. (2025) who noted that individuals with endometriosis engage in food restriction and dietary changes to manage endometriosis-related inflammatory symptoms such as bloating rather than for weight control alone.

The following limitations should be considered when interpreting the findings of this study. As the study used a cross-sectional design, it cannot determine causation between endometriosis-related variables (e.g. symptom burden, endometriosis-related pain), body compassion, and body image. Findings may also be limited by the self-reported endometriosis diagnosis, which was not confirmed through provision of medical records. However, recent evidence exploring the validity of self-reported endometriosis across four samples showed >70% accuracy with medical records (Shafrir et al., 2021). Notably, the online survey platform allowed for data to be gathered from a large community-based sample to obtain a representative sample rather than being limited to a single clinical setting. An additional limitation is that the sample comprises participants residing in more economically developed countries (MEDCs), with further research required to determine whether cultural factors may influence body compassion and body image in endometriosis. The current study did not use a measure of social desirability and therefore it is not possible to determine whether the body-related responses were influenced by social desirability. However, the use of an anonymous online survey for data collection may mitigate concerns relating to social desirability, with online survey use suggested to lead to more accurate self-disclosure of sensitive or health-related experiences (Rickwood and Coleman-Rose, 2023). Further research investigating the relationship between common humanity and body image in endometriosis is recommended to further understand the current finding that higher levels of common humanity are associated with a more negative body attitude. Lastly, further research is recommended that assesses the role that psychiatric comorbidity may have in influencing body-related outcomes in endometriosis as the current study did not assess for psychiatric comorbidity.

The current research has identified the importance of assessing and managing the negative influence endometriosis may have on an individual’s body image by identifying key symptoms including endometriosis-specific pain, nonmenstrual abdominal pain, nausea, dyspareunia, and bloating. Specifically, assessment of negative appreciation of body size and lack of body familiarity are hypothesized to be potential areas of clinical focus and may highlight the need for psychological therapy referral. Psychological therapy that encompasses compassion-focused skills, particularly in the areas of defusion and acceptance are suggested where concerns regarding body image are noted (Pehlivan et al., 2024; Skinner and Kuijer, 2025). Preliminary evidence suggests that body compassion, with a focus on defusion and acceptance, may act as a protective buffer, reducing the potential negative influence of endometriosis on an individual’s body image. Future interventional research is recommended to determine the clinical utility of body compassion therapy for addressing body image concerns in individuals with endometriosis.

## Supplemental Material

sj-docx-1-hpq-10.1177_13591053251401720 – Supplemental material for Correlates of body image in individuals with endometriosis: The role of body compassion and endometriosis-related symptoms Supplemental material, sj-docx-1-hpq-10.1177_13591053251401720 for Correlates of body image in individuals with endometriosis: The role of body compassion and endometriosis-related symptoms by Leesa Van Niekerk, Cecilia Hoi Man Ng, Louise Gibson, Rebecca O’Hara, Antonina Mikocka-Walus, Kimberley Norris, Mathew Leonardi, Mike Armour and Subhadra Evans in Journal of Health Psychology

sj-docx-2-hpq-10.1177_13591053251401720 – Supplemental material for Correlates of body image in individuals with endometriosis: The role of body compassion and endometriosis-related symptoms Supplemental material, sj-docx-2-hpq-10.1177_13591053251401720 for Correlates of body image in individuals with endometriosis: The role of body compassion and endometriosis-related symptoms by Leesa Van Niekerk, Cecilia Hoi Man Ng, Louise Gibson, Rebecca O’Hara, Antonina Mikocka-Walus, Kimberley Norris, Mathew Leonardi, Mike Armour and Subhadra Evans in Journal of Health Psychology
