# Supplementary material for: Correlates of body image in individuals with endometriosis: The role of body compassion and endometriosis-related symptoms
Source: J Health Psychol. 2026 Jan 15;31(11):4298–313. doi: 10.1177/13591053251401720 (PMC13490647; doi:10.1177/13591053251401720)
Supplement: Supplementary file 1 [file sj-docx-1-hpq-10.1177_13591053251401720.docx]

**Supplementary Table 1.**  *Average Distress Ratings Associated with Endometriosis-Related Symptoms Experienced in the Previous Four-Week Period.*

| Endometriosis-related symptom | *n* | *M* (*SD*) |
| --- | --- | --- |
| Difficulty conceiving  Heavy menstrual bleeding  Dysmenorrhea (painful periods)  Fatigue  Dyspareunia  Abdominal pain, when not menstruating  Pain after sexual intercourse  Pain associated with bowel movements  Vulva pain  Clitoral pain  Nausea  Bloating  Pain associated with urination  Lower back pain, when not menstruating  Intermenstrual bleeding  Constipation  Diarrhoea | 31  87  141  203  129  219  103  198  77  26  173  223  102  200  67  174  132 | 2.68 (.79)  2.32 (.76)  2.32 (.73)  2.05 (.79)  2.00 (.76)  2.00 (.81)  1.96 (.82)  1.90 (.86)  1.83 (.85)  1.81 (.85)  1.81 (.85)  1.79 (.92)  1.71 (.80)  1.68 (.79)  1.66 (.96)  1.59 (.82)  1.44 (.78) |

*Note:* Participants indicated the number of endometriosis-related symptoms experienced (out of a specified 17) in the four weeks prior to survey completion and the level of distress associated with these symptoms (0 = Not Distressing to 3 = Extremely Distressing).
