# Supplementary material for: Correlates of body image in individuals with endometriosis: The role of body compassion and endometriosis-related symptoms
Source: J Health Psychol. 2026 Jan 15;31(11):4298–313. doi: 10.1177/13591053251401720 (PMC13490647; doi:10.1177/13591053251401720)
Supplement: Supplementary file 2 [file sj-docx-2-hpq-10.1177_13591053251401720.docx]

**Supplementary Results File 1**

**Menopausal Status and Study Outcomes**

To investigate whether menopausal status influenced body compassion, body image, or endometriosis-related symptom burden and associated distress, independent samples t-tests were conducted. No statistically significant differences were noted between non-menopausal individuals (*n =* 207) and those experiencing symptoms of perimenopause, surgical, or medical menopause (*n =* 54) in the current sample (see Supplementary Results Table 1).

*Supplementary Results Table 1. Menopausal Status, Body Compassion, Body Image, and Endometriosis-Related Symptom Burden and Distress.*

|  | *M (SD)* | | *F* | *Sig.* | *t* | *df* |
| --- | --- | --- | --- | --- | --- | --- |
|  | Non-Menopause | Per/Surgical/Medical Menopause |  |  |  |  |
| Body Attitude Test  Negative appreciation of body size  Lack of body familiarity  General body dissatisfaction  Body Compassion  Defusion  Common humanity  Acceptance  Endometriosis-related symptom distress  Endometriosis-related symptom burden | 49.32 (15.01) | 51.67 (15.17) | .32 | .574 | -1.02 | 259 |
|  | 17.28 (7.26) | 18.64 (7.09) | .01 | .936 | -1.24 | 259 |
|  | 14.33 (4.55) | 15.35 (4.74) | .44 | .506 | -1.46 | 259 |
|  | 11.48 (5.03) | 11.94 (4.87) | .17 | .684 | -.61 | 259 |
|  | 58.55 (14.40) | 62.72 (13.53) | 1.02 | .313 | -1.92 | 259 |
|  | 22.08 (7.40) | 24.07 (7.38) | .01 | .986 | -1.76 | 259 |
|  | 23.12 (7.53) | 25.46 (6.86) | .67 | .412 | -2.08 | 259 |
|  | 13.35 (4.77) | 13.18 (4.10) | 1.79 | .182 | .24 | 259 |
|  | 1.82 (.51) | 1.85 (.59) | 3.54 | .061 | -.46 | 259 |
|  | 8.96 (3.04) | 8.31 (3.23) | .16 | .687 | 1.36 | 259 |
